# Whole-Genome Sequence of Burkholderia pseudomallei Strain HNBP001, Isolated from a Melioidosis Patient in Hainan, China

**DOI:** 10.1128/MRA.00471-19

**Published:** 2019-09-05

**Authors:** Xun Kang, Zengzhen Fu, Mamy Jayne Nelly Rajaofera, Chenchu Li, Nan Zhang, Lin Liu, Qinghui Sun, Chuizhe Chen, Sufang Dong, Hao Xiu, Xiuyu Ou, Chengli Liu, Hua Pei, Na He, Qianfeng Xia

**Affiliations:** a Key Laboratory of Tropical Translational Medicine of Ministry of Education and School of Tropical Medicine and Laboratory Medicine, Hainan Medical University, Haikou, Hainan, China; b Department of Pediatrics, Hainan Maternity and Child Healthcare Hospital, Haikou, China; Loyola University Chicago

## Abstract

Here, we report the complete genome sequence of Burkholderia pseudomallei HNBP001, an epidemic strain isolated from a melioidosis patient with pneumonia in Hainan, China.

## ANNOUNCEMENT

Burkholderia pseudomallei is a Gram-negative bacterium that causes melioidosis, a serious human and animal infection commonly reported in parts of Africa, South America, and Asia (1, 2). This organism was possibly used as a biological weapon (3). The limited treatment options combined with the difficulty in diagnosis and the current lack of a vaccine make this organism important to public health in tropical regions worldwide (4). In China, melioidosis is primarily reported in the southeast coastal regions, including Hainan, Guangdong, Fujian, and Taiwan (5, 6). The organism is widespread in water and soil of its regions of endemicity (7). Infection is commonly acquired via contact with a contaminated environmental source (8). Deciphering whole-genomic sequences provides insight into the pathogen’s phylogeny or diversity and the biogeographical contribution of virulence; however, the majority of the currently available sequences of B. pseudomallei are from strains isolated in northern Australia or Thailand (9, 10). Here, we report the genome sequence of B. pseudomallei strain HNBP001, isolated from the blood of a melioidosis patient with pneumonia using a previous method (6). Sampling procedures were approved by the ethics committee of the Hainan Medical University.

For the draft genome sequence, B. pseudomallei HNBP001 was grown on a Luria-Bertani (LB) agar plate with gentamicin for 24 h at 37°C (11). Genomic DNA was extracted using a bacterial DNA extraction kit (Omega Bio-tek, USA) and then sequenced using the PacBio RS II platform at the Beijing Genomics Institute (BGI) in Shenzhen, China. PacBio subreads with a length of <1 kb were removed in order to obtain more accurate and reliable subreads in the subsequent bioinformatics analysis. For PacBio sequencing, approximately 10 g of DNA was fragmented to 10 to 20 kbp using the g-TUBE apparatus (Covaris). The sequencing library was constructed using the SMRTbell template prep kit 1.0 according to the PacBio 20-kb library protocol. Sequencing was performed on the PacBio RS II instrument in one single-molecule real-time (SMRT) cell (v3) for 6 h. Illumina libraries were constructed with 270-bp inserts using standard methods. The paired-end sequencing library was prepared using a Genomic DNA prep kit (Qiagen, USA) and then sequenced using an Illumina HiSeq 4000 platform. A total of 1,115 Mb of raw data were produced from 7,438,126 150-bp reads. After filtering out low-quality reads with SOAPnuke using default parameters (12), we obtained 1,024 Mb clean short reads. The genome was assembled with the Hierarchical Genome Assembly Process 3 (HGAP3) (13) and Quiver in SMRT analysis v2.3.0, GATK (https://www.broadinstitute.org/gatk/), SOAPsnp (14), and SOAPindel (15) to correct small indels, resulting in 2 contigs representing the 2 chromosomes in B. pseudomallei HNBP001. The genome was annotated with Glimmer3 v3.02 using default parameters (16). There were 59,381 polymerase reads after filtering. The data size of all the polymerase reads was 877,413,219 bp, and their average length was 14,775 bp. The average quality of all polymerase reads was 0.68 read quality (RQ). There were 102,965 filtered subreads with a total data size of 873,175,857 bp. The average length of the subreads was 8,480 bp with an *N*_50_ value of 10,525 bp. The average quality of all subreads was 0.86 RQ.

The B. pseudomallei HNBP001 genome consists of two chromosomes. The newly sequenced complete genome sequence of HNBP001 comprises a large chromosome and a small chromosome of 7,163,595 bp in total. Chromosome 1 is 3,800,466 bp in size (GC content, 68.04%) with 3,807 protein-coding sequences, 6 rRNA genes, and 45 tRNA genes. Chromosome 2 is 3,363,129 bp in size (GC content, 68.32%) with 3,342 protein-coding sequences, 6 rRNA genes, and 14 tRNA genes. These numbers are similar to those of previously published genome annotations of B. pseudomallei (17, 18). The genome sequence of strain HNBP001 will serve as a reference for additional assemblies of B. pseudomallei isolates from China.

### Data availability.

The finished annotated sequences of the two chromosomes of B. pseudomallei have been deposited at NCBI GenBank under the accession numbers CP038805 (chromosome 1) and CP038806 (chromosome 2). The raw data were deposited in the Sequence Read Archive (SRA) for the PacBio RS II (SRA number SRR9027110) and Illumina (SRA number SRR9691870) read data.
